# Humidity-Induced Charge Leakage and Field Attenuation in Electric Field Microsensors

**DOI:** 10.3390/s120405105

**Published:** 2012-04-19

**Authors:** Haiyan Zhang, Dongming Fang, Pengfei Yang, Chunrong Peng, Xiaolong Wen, Shanhong Xia

**Affiliations:** 1 State Key Laboratory of Transducer Technology, Institute of Electronics, Chinese Academy of Sciences, Beijing 100190, China; E-Mails: zhanghyie@yahoo.com.cn (H.Z.); fangdm@pku.edu.cn (D.F.); yang330650591@126.com (P.Y.); chunrong_p@163.com (C.P.); 15901150155@163.com (X.W.); 2 Graduate School of the Chinese Academy of Sciences, Beijing 100049, China

**Keywords:** electric field sensor, charge leakage, electric field attenuation, water film thickness, MEMS

## Abstract

The steady-state zero output of static electric field measuring systems often fluctuates, which is caused mainly by the finite leakage resistance of the water film on the surface of the electric field microsensor package. The water adsorption has been calculated using the Boltzmann distribution equation at various relative humidities for borosilicate glass and polytetrafluoroethylene surfaces. At various humidities, water film thickness has been calculated, and the induced charge leakage and field attenuation have been theoretically investigated. Experiments have been performed with microsensors to verify the theoretical predictions and the results are in good agreement.

## 1. Introduction

Electrostatic field measurement has been the subject of a lot of research over the past half century [1]. Although many devices have been developed [2–14], the variable capacitance field mills have always been the most prevalent device used in the measurement of electric field [1–3]. However, with the development of micro-electro-mechanical system (MEMS) technology, more and more researchers have paid attention to electric field microsensors (EFMS). In 1991, Hsu first reported the prototype of a micro-miniature, non-contacting electrostatic voltmeter (ESV) [15]. In 2001, Horenstein reported a miniature electrostatic field mill which was fabricated using the MEMS silicon surface micromachining process [1]. In 2003, Riehl reported high performance MEMS EFMS [16]. In 2004, Gong presented the design and optimization of two kinds of novel miniature off-plane vibrating EFMS based on MEMS technology [17]. In 2005, Shafran exploited a MEMS-based electric field meter and a closed feedback loop circuit was used to maximize its displacement and the magnitude of the dynamic current [18]. In 2006, Peng reported a kind of electrostatic comb-driven EFMS based on PolyMumps MEMS technology [19]. In the same year, Chen reported a thermally driven micro-electrostatic field meter [20]. In 2010, a comb-driven EFMS grounded beam moving laterally between positive and negative sense electrodes was investigated in [21] by Peng. According to the latest results reported by Peng, the minimum detectable electric field with EFMS is better than 50 V/m in ambient air, which meets the requirement of most electric field measurements cases. However, all the above sensors neglected the effect of humidity in actual measurement environment, hence, the performance of the sensor will degrade in the long-term extraventricular measurement.

The aforementioned EFMS have a similar operation principle as field mills. Generally, the EFMS has shutter and sensing electrodes fabricated in the same chip. As the shutter oscillates back and forth, it covers the sidewalls or top surface of the positive or negative sensing electrodes, causing a differential AC current, and then the external electric field can be calculated from the current. However, unlike the traditional large volume field mill, an EFMS is a small exact electronic device, that should be packaged to protect it from external physical injury and ensure its operation in the atmosphere. Nevertheless, its stability and reliability still remain the key problems. Any environmental change, such as temperature and humidity, will influence the stability of the measurement. Typically, the charge leakage through the pin and package cap causes a decline in the stability of EFMS. These two parts' charge leakage occur mainly because of surface contaminants, especially the water film adsorbed in the package surface due to the environmental humidity.

**Figure 1.**
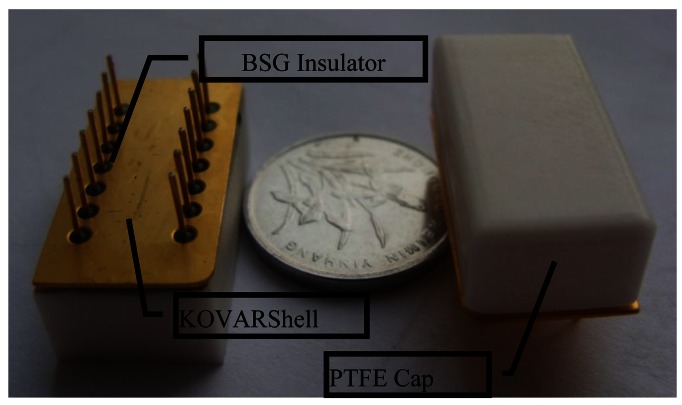
Photograph of an EFMS package with PTFE cap.

In this work, a 14-pin dual in-line (DIP14) integrated KOVAR shell was adopted as the support of the EFMS die. The pin and the KOVAR shell are isolated by a Borosilicate Glass (BSG) ring. Metal is not suitable to be the top surface of EFMS package for it could form a shield from the external electric field and prevent the sensing component from detecting the external electric field. Here, polytetrafluoroethylene (PTFE) was chosen as the material of the cap of package because of its high resistivity and good chemical stability, which could resist a corrosive gas. A photograph of a packaged EFMS is shown in Figure 1.

With the increase of the environmental humidity, the electrical conductivity in the surface of the PTFE cap and BSG also increases due to the increase of the thickness of the existent water film. The Boltzmann distribution equation is adopted in this paper to ascertain the water film thickness under various environmental humidity conditions. If the conductivity of the water film is given, the resistivity of the charge leakage channel can be calculated, and then the magnitude of charge leakage can also be calculated. Meanwhile, the attenuation time constant and magnitude of electric field with environmental humidity in the inner package can be deduced. The parameters used in this paper are listed in Table 1. The packaged EFMS was tested under various environmental humidities, and the results compared with the calculations to verify the calculation method used in this paper.

## 2. Modeling and Calculation of Charge Leakage and Field Attenuation

### 2.1. Calculation of the Water Film Thickness

Due to the molecular interaction energy of water in the surface and in the air, *μ_1_^i^* and *μ_2_^i^*, the molecular molar concentrations in the two districts at equilibrium, *X_1_* and *X_2_*, fit the Boltzmann distribution [22]. This can be expressed by:

(1) $$\mu_{1}^{i}+kT\log X_{1}=\mu_{2}^{i}+kT\log X_{2}$$

where *k* is the Boltzmann constant, 1.38 × 10^−23^ J/K, *T* is the thermodynamic temperature.

Then the Equation (1) can be converted into Equation (2):

(2) $$X_{1}=X_{2}\exp\left[\overset{-\left(\mu_{1}^{i}-\mu_{2}^{i}\right)}{\;}/\underset{kT}{\;}\right]$$

Assuming the water molecular concentration in the air is *ρ_1_*, in the surface is *ρ_0_*, since *μ_1_^i^* − *μ_2_^i^* = −*mgD*, then Equation (3) can be written as:

(3) $$\rho_{1}=\rho_{0}\exp(-\text{mgD}/\text{kT})$$

where *m* is the water molecular mass, *g* is the gravitational acceleration, *D* is the thickness of the water film.

The water molecular number density with certain pressure and temperature also means vapor pressure. Assuming the saturated vapor pressure is *P_0_*, air vapor pressure is *P*, which put up as adhesive pressure at the surface of the water film and the air.

Then following equation can be obtained:

(4) $$P=P_{0}\exp\left(-\text{mgD}/kT\right)$$

On the other hand, the equilibrium water film thickness that can counteract the adhesive pressure can be expressed by:

(5) P=ρgD

while:

(6) $$P=A/6\pi D^{3}$$

where *A* is the Hamaker constant, for water and PTFE interacting surface, *A_PTFE_* = 0.29 × 10^−20^ J [22], for water and BSG interacting surface, *A_BSG_* = 2.0 × 10^−20^ J.

and:

(7) m=ρv

where *ν* is the volume of the single water molecule.

Hence:

(8) $$\overset{P}{\;}/\underset{P_{0}}{\;}=\exp\left[-Am/6\pi D^{3}\rho kT\right]$$

In addition, environmental relative humidity can be expressed by:

(9) $$RH=\overset{P}{\;}/\underset{P_{0}}{\;}$$

Therefore, after combining Equation (8) and Equation (9), the thickness of the water can be written as:

(10) $$D=\sqrt[{3}]{\frac{-Am}{6\pi \rho kT\ln(RH)}}$$

**Figure 2.**
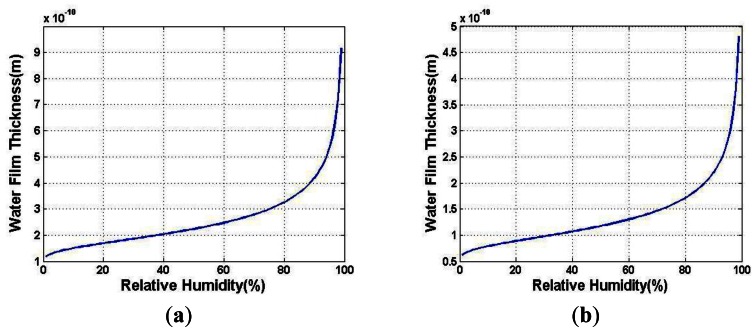
(**a**) Calculated water film thickness on BSG; (**b**) Calculated water film thickness on PTFE.

Based on the above deduction, the adsorbed water film thickness in the surface of solid can be expressed with the function of relative humidity [23]. Substituting the value of the parameters, the water film thickness in the surface of BSG (*D_1_*) and PTFE (*D_2_*) are plotted in Figure 2, respectively.

### 2.2. Charge Leakage through the Water Film Adsorbed in the Surface of BSG

Due to the water molecule absorbability of BSG, it would form a charge leakage channel by water molecules adsorbed on the surface with the increase of environmental humidity. The account of the various actual water cladding shapes will limit the use of water film thickness calculation model, the physical model of water cladding in the surface of BSG was simplified and the thickness of water film was assumed to be uniform. Then the charge leakage channel can be treated as ring-shaped resistor. Then the resistor *R_w_* of the water film can be calculated by the following equation [24]:

(11) $$R_{w}=\overset{\rho_{w}r}{\;}/\underset{S_{w}}{\;}$$

where *ρ_w_, _r_*_,_and *S_w_* represents the resistivity, radius and cross section area of water film, respectively.

EFMS mentioned in this paper has two output pins, and beside each output pin there is a driving signal pin. Hence, the resistors caused by surface water film can be seemed as the parallel resistor, and the value is reduced by half:

(12) $$R_{w}=\frac{1}{2}\int_{r_{1}}^{r_{2}}\frac{\rho_{w}}{2\pi D_{1}r}dL=\frac{1}{2}\frac{\rho_{w}}{2\pi D_{1}}\ln\left(\overset{r_{2}}{\;}/\underset{r_{1}}{\;}\right)$$

where *D_1_* is the thickness of water film, *r_1_* and *r_2_* are the inner and outer radius of the ring-shaped water film.

The driving signal coupling to sensing output pin through the water film resistor forms a leakage current, then translates to the voltage signal through the feedback resistor of the pre-processing circuit, after the amplification of the amplifying circuit, the sensing voltage signal is converted to digital signal. The final sensor output drift can be written as:

(13) $$\Delta V_{o}=-A_{c}\frac{V_{d}R_{f}}{R_{w}}=-A_{c}\frac{2\pi D_{1}V_{d}R_{f}}{\rho_{w}\ln(\frac{r_{2}}{r_{1}})}$$

where *A_c_* is the transmission gain of the amplifying circuit, *V_d_* is the voltage of driving signal, and *R_f_* is the feedback resistor of pre-processing circuit.

By combination of Equation (10) and Equation (13), the zero point drift of EFMS caused by humidity can be obtained.

### 2.3. Electric Filed Attenuation Caused by the Water Film in PTFE Cap

**Figure 3.**
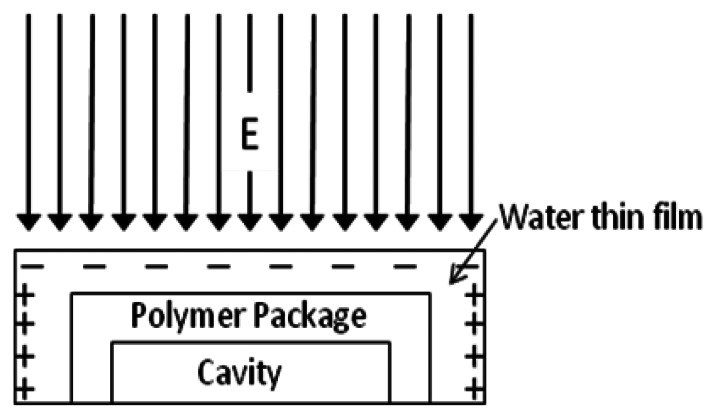
Sketch of electric field attenuation.

PTFE itself is hydrophobic, but it becomes hydrophilic if its surface is destroyed or contaminated. Especially for cap surfaces made by machining, the water molecules in the air are prone to be adsorbed onto the PTFE cap of the EFMS package and form a thin water film whose thickness is dependent on the humidity of the surrounding air. By the theory of liquid charging, the charge in the water film will transfer along the direction of the external electric field. Finally, the additive inverse electric field caused by charge motion in the water will neutralize the external electric field. For the EFMS die localized in the package, this neutralization causes an electric field attenuation. The mechanism of electric field attenuation can be explained by the sketch of Figure 3.

The time constant of the attenuation is decided by the conductivity of the water film, in other words, the thickness of water film. On the other hand, the thickness is related to the environmental relative humidity and the property of the package material.

The package was supposed to be an isolated linear homogeneous medium, and the dielectric constant is *ε*, conductivity is *σ*, the volume charge density is *ρ_v_*. As mentioned above, the electric field in the film will neutralize the external electric field. Hence, there is current equilibrium in the water film, and it can be expressed as:

(14) $$\nabla J+\frac{\partial \rho_{v}}{\partial t}=0$$

where *J* is the current density.

Since *J* = *óE*, Equation (14) becomes:

(15) $$\sigma \nabla E+\frac{\partial \rho_{v}}{\partial t}=0$$

Then substituting *ρ_v_*/*ε* for ∇*E*:

(16) $$\frac{\partial \rho_{v}}{\partial t}+\frac{\sigma \rho_{v}}{ɛ}=0$$

It is obvious that this is a one order differential equation about *ρ_v_*:

(17) $$\rho_{v}=\rho_{c0}e^{-(\overset{\sigma }{\;}/\underset{ɛ}{\;})t}$$

where *ρ_c0_* is the volume charge density at the time *t* = 0.

From Equation (17), it can be concluded that the equilibrium process will follow the exponential function. It is also obvious that the ratio of *ε* and *ó* has the dimension of time, therefore, it is called attenuation time constant:

(18) $$\tau =\frac{ɛ}{\sigma }$$

The attenuation time was used to estimate the speed to reach inner electric field equilibrium. From the above Equation, it is obvious that the greater the conductivity is, the sooner the equilibrium time will be.

Then the charge quantities *Q* in the PTFE cap at time *t* can be expressed as:

(19) $$Q=\rho_{v}WLD_{2}$$

where *W* and *L* are the width and length of the PTFE cap, respectively; *D_2_* is the thickness of water film on PTFE cap surface.

Because the water film is only a few nanometers, hence, the free charge in the water film can be treated as plane distribution, and the plane charge density *ρ_s_* can be expressed as:

(20) $$\rho_{s}=\frac{Q}{WL}=\rho_{v}D_{2}$$

Then the enantiomorphous electric field in the sensor E′ caused by the free charge in the top surface of cap can be calculated as:

(21) $$E^{\prime }=-\frac{\rho_{s}}{4\pi {ɛ}_{0}}\int_{s}\frac{\left(r-r_{i}^{\prime }\right)}{{\left|r-r_{i}^{\prime }\right|}^{3}}ds^{\prime }$$

where *S*′ is the top surface of PTFE cap, *r* and *r_i_* are the distances from top surface to sensor, here, *r* = 0.

Because the size of the PTFE cap is given, $\int_{s}\frac{\left(r-r_{i}^{\prime }\right)}{{\left|r-r_{i}^{\prime }\right|}^{3}}ds^{\prime }$ can be seemed as constant, in this paper we predigest it as *C*. Then the net electric field on the sensor is the synthetical field of the external and enantiomorphous electric field:

(22) $$\begin{array}{c} E_{\text{total}}(t)=E+E^{\prime }\\ =E-\frac{\rho_{c0}e^{-\left(\frac{\sigma }{\tau }t\right)}D_{2}C}{4\pi {ɛ}_{0}} \end{array}$$

According to Equation (22), it can be concluded that the sensor output in the certain electric field attenuated in the exponential function.

## 3 Results and Discussion

To confirm the calculation model of charge leakage through BSG, a KOVAR DIP14 packaged sensor was placed in a software controlled humidity box, and the PEFE cap was covered by grounded conductive tape to shield it from the external electric field. Hence, the attenuation effect induced by humidity can be neglected, and the sensor output is only related to the charge leakage caused by the water film in the surface of BSG. Limited by the program controlled humidity box, the humidity ranges from 20% to 95% in 5% intervals, and the temperature was set at 20 °C. The measuring process is listed below:

1. Set the humidity value of the box.
2. Run the box, wait the reading of humidity meter in the box to the set value.
3. Wait 2 hours to ensure the environmental humidity is stable enough.
4. Sample 100 points of zero output, and record the mean value.
5. Repeat steps (1)∼(5), from 25% to 95%.

**Figure 4.**
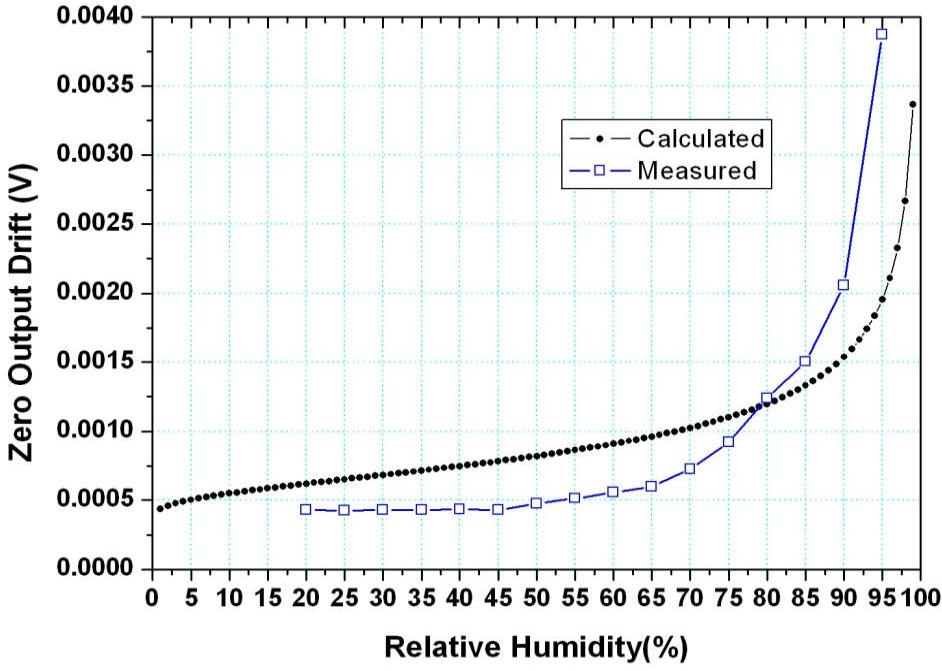
Sensor zero drift caused by charge leakage through water film in the surface of BSG.

For comparison, the calculated and measured results are shown in Figure 4.

As shown in Figure 4, the measured data and the calculated data has the same trend as a whole, since both of them increased exponentially with the increase of humidity. However, there still are some errors between them. When the humidity was below about 80%, the measured data is a little smaller than those calculated, and the situation is reversed while the relative humidity is above 80%. There are two reasons for these errors.

Firstly, when the humidity is below 80%, the calculated water film thickness is smaller than the water molecule diameter, hence, the water molecule in the surface of BSG is not continuous, in a practical case, the water film is not a continuous film, and it can't be an effective leakage channel. In the calculation model, in spite of the thickness of water film being smaller than the diameter of the water molecule, the film is still deemed to be continuous, and hence, it has conductivity. In a word, at the lower environmental humidity (<80%), the neglection of discontinuity in the model is the main source of the error between the measured and calculated results, and the maximum error is about 0.3 mV at 60%. R.H.

Secondly, when the humidity is above 80%, the deviation is mainly caused by the error of the water film thickness between the actual and calculated. The calculation assumed the water film is uniform, but the actual water film has a certain roughness. The water molecule is adsorbed in the concave shape first and then the film is formed. Hence, the actual thickness of the water film is larger than the calculated one, and so is the conductivity. Hence, we can conclude that the actual zero output drift is larger than that of the calculated one, the error between them becoming larger as the humidity increases. More detailed reasons for this will be analyzed in the future.

**Figure 5.**
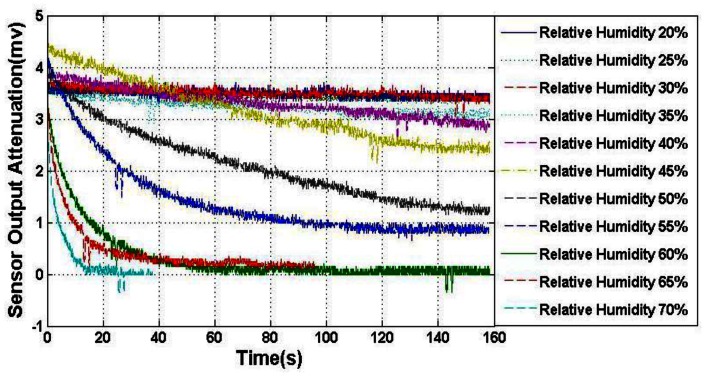
Sensor output caused by field attenuation before package surface processing.

In order to verify the calculation model of external electric field attenuation, a humidity experiment was also carried out. For the reason that PTFE cap surface cleanliness can't be measured accurately, limited by measuring technique, this test only allows a trend analysis. The setup is similar to the former experiment, but the KOVAR shell was sealed to protect the MEMS sensor die from air moisture, and PTFE cap surface was kept unprocessed. The standard electric field generator is also placed in the box. Humidity was varied from 20% to 70% with an interval of 10%, the electric field attenuation was measured under certain electric field strength (50 kV/m), and the results are shown in Figure 5. It can be seen that the electric field attenuation became severe with the increase of humidity, and the attenuation time constant became shorter, becoming only about 20 s under 70% humidity.

**Figure 6.**
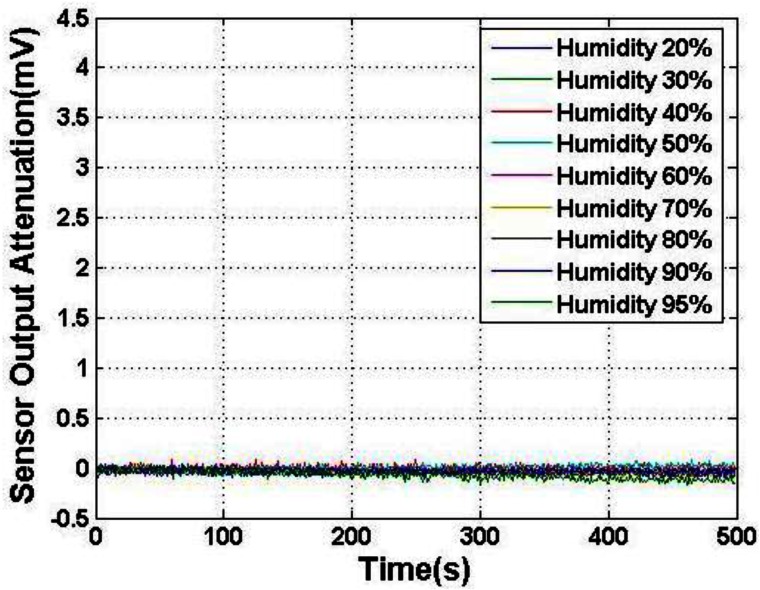
Sensor output caused by field attenuation after package surface processing.

To alleviate the electric field attenuation, the PTFE cap was cleaned. The cleaning solution contained H_2_SO_4_, H_2_O_2_, deionized water (the proportion is 1:3:17). The bath temperature is 70 °C. The PTFE cap was rinsed in the cleaning solution for 2 h, then stuck to the KOVAR shell and baked for 15 min at 70 °C. The test result of the attenuation is shown in Figure 6, whose vertical axis range is kept the same as Figure 5. The electric field attenuation effect was insignificant and was barely discernible, and there is only circuit noise while the environmental relative humidity is below 90%. We can conclude that the electric field attenuation can be neglected. The attenuation time constant was considered infinite. The attenuation was observed to be rapid when the humidity was higher than 90%, as expected, and the attenuation time constant was about 23,000 s. Therefore, we can choose the right time to read the output of MEMS EFMS with the attenuation time constant and calculate the real electric field strength according to the attenuation factor at various humidity. Because the PTFE cap was fabricated by machining and the roughness was poor, the splattered small concave beads at RH < 90% became consecutive and formed an effective charge leakage channel, hence, the conductivity *σ* became larger than that of humidity below 90%. On the whole, with the cleaning of the PTFE cap, the water film deposited on it was split to small beads, so the conductivity of the film was minimized. Hence, the electric field attenuation time constant was nearly infinite, and the EFMS performance stability was improved.

## 4. Conclusions

We have theoretically and experimentally studied two mechanisms whereby the environmental humidity affects the electric field measurement, *i.e*., leakage charge from package pins and field attenuation caused by the water film on the package surface. The calculation of the water film thickness in various humidity environments is presented. The finite leakage resistance can be derived after the water film thickness calculation, and then be used for calculation of the leakage charge and the electric field attenuation. The attenuation time constant can also be calculated with the dielectric constant of water. Experiments are carried out to verify the feasibility of the calculation, and the experimental results agree well with those calculated.

## Figures and Tables

**Table 1.** Parameters used in the calculation.

Parameter	Explanation	Value
*ρ*	Density of water	1 × 10^3^ kg/m^3^
*m*	Mass of water molecule	2.99 × 10^−26^ kg
*k*	Boltzmann constant	1.38 × 10^−23^ J/K
*T*	Room temperature, 25 °C	298 K
*ρ_w_*	Resistivity of water	3.2 × 10^4^ Ωm
*r_1_*	Inner readius of BSG ring	1 mm
*r_2_*	Outer readius of BSG ring	2 mm
*A_c_*	Gain of preprocessing circuit	80
*V_d_*	DC component of driving signal	8 V
*σ*	Conductivity of water	0.31 × 10^−4^ S/m
*ε*	Dielectric constant	78.36 F/m
